# Mortality of Philadelphia for the Quarter Ending April 1, 1857

**Published:** 1857-05

**Authors:** Wilson Jewell


Art. VI.—*Mortality of Philadelphia, for January, February, and March, 1857, collated from the Health Office Record.* By Wilson Jewell, M.D.

During the first quarter of the present year, including a period from January 3d to March 28th, the deaths in our Consolidated City, recorded at the Health Office, have amounted to 3042.

This total, is an increase of 232, or nearly four per cent., over those for the first quarter of 1856.

Included in the above deaths are 430 from adventitious causes. Among these will be found 129 still-born children.

The excess of male deaths is equal to 4·84 per cent. Of the deaths under twenty, or among children, there were 1913; of these, 1039 were boys, and 874 girls, showing an excess of 8·62 per cent. among male children.

The deaths among children under five years of age, numbered 1433, equal to 49 per cent., or nearly one half of all the deaths for the quarter, exclusive of the still-born, which amounted to 129.

The first week in February records the highest number of deaths weekly, viz., 297; while the last week in March gives the lowest, viz., 201.

The deaths from zymotic diseases have been 754. In the month of January they were 345; in February 237, and in March only 172—showing a falling off of one half below those of January. This reduced mortality is owing in a great measure to the decline in the deaths from scarlet fever.

It will be noted, that nearly one half the deaths under this head, are registered from scarlet fever, viz., 425! nearly equal to the mortality for the previous quarter, which was 438. This fatal epidemic, which has been prevalent for the last nine months, is at present disappearing from our midst.

Measles, which contributes 53 to this class of deaths, is still prevalent and quite fatal. In connection with this disease, there has been in existence a form of eruption which resembles measles, but without catarrh, and with very little if any febrile reaction. It is of short duration, seldom lasting over four days, and the rose-colored eruption beginning to fade generally on the second day after its appearance. We have considered it as Roseola, while some have called it French measles (?), and others, *Rubeola sine Catarrho*. If it is, indeed, a mild variety of measles, it does not exempt the patient from an attack of the febrile form.

In three instances, we have known genuine measles to follow this eruption within one month, and in a number of families we have met with it, where we had attended the same children for measles within the last two years.

We have witnessed three cases of this roseola in adults, but in each they were unattended with active febrile symptoms, or the phenomena of ordinary catarrh.

Croup has been, as usual during the winter season, of very frequent occurrence. The deaths during the quarter amounted to 89. We have seen several cases of croup following closely upon an attack of measles.

Small-pox has been on the increase during the last three months. The deaths have amounted to 32; of this number, 23 were of children under five years of age,—in all probability, unprotected by vaccination. There are also recorded three deaths from varioloid, under two years of age (?).

Of the deaths under the head of uncertain or general seat, which amount to 396, we have to allude to the large number from dropsy, viz., 91! More than double those for the same quarter of 1856. Of these deaths, 38 were under ten years of age, and in all probability, a majority of them were the sequence of scarlet fever, and should properly be classified with that disease, or else recorded as "dropsy, the result of scarlet fever."

The diseases of the nervous system (the brain and spinal marrow), usually contribute a large proportion of the deaths. In this instance, they furnish 486 towards the total. Of this sum, 141 were from convulsions, the most of which deaths took place before the fifth year, viz., 124.

One hundred and eighty-eight (188) of these deaths are recorded from inflammation, congestion, and dropsy of the brain. A very large proportion of the deaths in this class, viz., 348, occurred in children under ten years of age.

The deaths under the head of "Organs of Respiration," are more numerous than those of any other class. They are set down at 764, or 26·22 per cent. of all the deaths exclusive of still-born.

Consumption of the lungs, in this class, furnishes 407 deaths, or nearly 14 per cent. of all the deaths for the quarter, exclusive of still-born.

Inflammation of the lungs, the next highest, contributed 175 deaths; of these, 105 had not reached their fifth year.

The deaths from the diseases of the organs of respiration, during this quarter, have increased 6·48 per cent. over those for the same quarter of 1856.

The diseases of the "Organs of Circulation" furnish 74 deaths during the quarter. Under the term, "Disease of the Heart," we find recorded 49 deaths, more than half of the whole number. The vagueness of this term, however, like many others we meet with in our bills of mortality, leave us in the dark, as to the precise character of the lesion of this organ, which the certificate of the medical attendant is expected to furnish.

The deaths enumerated under the head of "Digestive Organs," amount to 151; of these 70 were from inflammation of the stomach and bowels, and 23 from inflammation of the peritoneum. 22 of the latter were among females.

Among the deaths from diseases of the "Organs of Generation," we find 33 from puerperal fever, and two from child-bed, making 35 deaths during the lying-in period. This is a greater mortality among women in the parturient state, than we have ever been called upon to record for a single quarter, since 1852.

TABLE No. 1.

*Deaths for the First Quarter of the Year* 1857, *classified.*

| | | | | Male. | | | Female. | | | |
|---|---|---|---|---|---|---|---|---|---|---|
| | Jan'y | Feb. | Mar | J. | F. | M. | J. | F. | M. | Total. |
| 1. *Endemic and Contagious Diseases.* | | | | | | | | | | |
| Zymotic or Epidemic, . . . . . | 345 | 237 | 172 | 186 | 129 | 107 | 159 | 108 | 65 | 754 |
| 2. *Uncertain or General Seat.* | | | | | | | | | | |
| Sporadic Diseases, . . . . . . . | 139 | 138 | 119 | 69 | 77 | 49 | 70 | 61 | 70 | 396 |
| 3. Nervous System, . . . . . . . | 174 | 166 | 146 | 95 | 96 | 79 | 79 | 70 | 67 | 486 |
| 4. Organs of Respiration, . . . . . | 266 | 241 | 257 | 139 | 119 | 129 | 127 | 122 | 128 | 764 |
| 5. " Circulation, . . . . | 25 | 23 | 26 | 16 | 13 | 12 | 9 | 10 | 14 | 74 |
| 6. Digestive Organs, . . . . . . . | 45 | 53 | 53 | 23 | 28 | 21 | 22 | 25 | 32 | 151 |
| 7. Urinary " . . . . . . | 7 | 5 | 2 | 5 | 5 | 2 | 2 | | | 14 |
| 8. Organs of Generation, . . . . | 16 | 21 | 16 | | | | 16 | 21 | 16 | 53 |
| 9. " Locomotion, . . . . | 5 | 5 | 2 | 4 | 3 | | 1 | 2 | 2 | 12 |
| 10. Integumentary System, . . . . | 1 | | | | | | 1 | | | 1 |
| 11. Old Age, . . . . . . . . . . | 13 | 13 | 19 | 2 | 5 | 9 | 11 | 8 | 10 | 45 |
| 12. External Causes, . . . . . . . | 25 | 25 | 28 | 20 | 15 | 19 | 5 | 10 | 9 | 78 |
| Still Born, . . . . . . . . . | 42 | 47 | 40 | 25 | 24 | 22 | 17 | 23 | 18 | 129 |
| Unknown, . . . . . . . . . | 36 | 21 | 28 | 21 | 11 | 16 | 15 | 10 | 12 | 85 |
| | 1139 | 995 | 908 | 605 | 525 | 465 | 534 | 470 | 443 | 3042 |

TABLE No. 2.

1. *Endemic and Contagious Diseases—Zymotic or Epidemic.*

| | Males. | Females. | Boys. | Girls. | Under 1 yr. | 1 to 2. | 2 to 5. | 5 to 10. | 10 to 15. | 15 to 20. | 20 to 30. | 30 to 40. | 40 to 50. | 50 to 60. | 60 to 70. | 70 to 80. | 80 to 90. | 90 to 100. | 100 to 110. | Total. |
|---|---|---|---|---|---|---|---|---|---|---|---|---|---|---|---|---|---|---|---|---|
| Cholera Infantum, . | 2 | | 2 | | 2 | | | | | | | | | | | | | | | 2 |
| Croup, . . . . . | 51 | 38 | 51 | 37 | 20 | 18 | 43 | 6 | 1 | | | 1 | | | | | | | | 89 |
| Diarrhœa, . . . . | 5 | 7 | | 3 | 2 | | 1 | | | | 2 | 1 | 1 | 1 | 2 | 1 | 1 | | | 12 |
| Dysentery, . . . . | 8 | 7 | 5 | 2 | 1 | 3 | | 2 | | 1 | 2 | 2 | 3 | 1 | | | | | | 15 |
| Erysipelas, . . . . | 21 | 14 | 7 | 6 | 7 | 1 | 2 | | 1 | 2 | 3 | 6 | 4 | 3 | 2 | 4 | | | | 35 |
| Fever, . . . . . | 1 | 2 | | | | | | | | | 2 | | | | | 1 | | | | 3 |
| " Bilious, . . . | 1 | 1 | | | | | | | | | | | 1 | 1 | | | | | | 2 |
| " Continued, . | 1 | 1 | | 1 | | | | | 1 | | 1 | | | | | | | | | 2 |
| " Gastric, . . | 2 | | 2 | | 1 | | 1 | | | | | | | | | | | | | 2 |
| " Intermittent, . | 3 | | 1 | | | | 1 | | | | | | 1 | | | 1 | | | | 3 |
| " Nervous, . . | 1 | 2 | | 1 | | | | 1 | | | 1 | 1 | | | | | | | | 3 |
| " Remittent,. . | 3 | 4 | 2 | 1 | 2 | | 1 | | | | | | 1 | 1 | 1 | 1 | | | | 7 |
| " Scarlet, . . . | 230 | 195 | 228 | 190 | 36 | 80 | 191 | 94 | 15 | 2 | 5 | 2 | | | | | | | | 425 |
| " Typhoid, . . | 26 | 16 | 12 | 5 | | 1 | 8 | 5 | 1 | 2 | 10 | 5 | 3 | 3 | 3 | 1 | | | | 42 |
| " Typhus, . . | 7 | 1 | 4 | 1 | 1 | | 2 | | 1 | 1 | 1 | | 1 | | | 1 | | | | 8 |
| Hooping-Cough, . . | 9 | 5 | 9 | 5 | 9 | 2 | 3 | | | | | | | | | | | | | 14 |
| Measles, . . . . . | 31 | 22 | 31 | 22 | 15 | 17 | 19 | 1 | 1 | | | | | | | | | | | 53 |
| Small-Pox, . . . . | 16 | 16 | 11 | 13 | 11 | 7 | 4 | 1 | 1 | | 5 | 2 | | 1 | | | | | | 32 |
| Syphilis, . . . . . | 1 | | 1 | | 1 | | | | | | | | | | | | | | | 1 |
| Varioloid, . . . . | 3 | 1 | 3 | 1 | 2 | 1 | | 1 | | | | | | | | | | | | 4 |
| | 422 | 332 | 369 | 288 | 110 | 130 | 276 | 111 | 22 | 8 | 32 | 20 | 15 | 11 | 8 | 10 | 1 | | | 754 |

2. *Uncertain or General Seat—Sporadic Diseases.*

| | Male. | Female. | Boys. | Girls. | Under 1 yr. | 1 to 2. | 2 to 5. | 5 to 10. | 10 to 15. | 15 to 20. | 20 to 30. | 30 to 40. | 40 to 50. | 50 to 60. | 60 to 70. | 70 to 80. | 80 to 90. | 90 to 100. | 100 to 110. | Total. |
|---|---|---|---|---|---|---|---|---|---|---|---|---|---|---|---|---|---|---|---|---|
| Abscess, . . . . . | 1 | 6 | | | | | | | | | 2 | | 2 | 3 | | | | | | 7 |
| Anasarca, . . . . . | 1 | | | | | | | | | | 1 | | | | | | | | | 1 |
| Cancer, . . . . . . | 9 | 18 | 1 | 1 | | | 1 | | | 1 | 1 | 3 | 6 | 8 | 2 | 3 | 2 | | | 27 |
| Cyanosis, . . . . . | 6 | 2 | 6 | 2 | 6 | 1 | 1 | | | | | | | | | | | | | 8 |
| Debility, . . . . . . | 48 | 43 | 24 | 20 | 39 | 1 | 3 | | | 1 | | 5 | 3 | 7 | 7 | 19 | 3 | 3 | | 91 |
| Disease of Leg, . . . | | 1 | | 1 | | | | 1 | | | | | | | | | | | | 1 |
| Throat, . . | 1 | | 1 | | 1 | | | | | | | | | | | | | | | 1 |
| Dropsy, . . . . . . | 42 | 34 | 28 | 13 | 4 | 3 | 18 | 13 | 2 | 1 | 2 | 5 | 3 | 7 | 8 | 7 | 3 | | | 76 |
| Fungus Hematodes, . | 1 | 1 | | | | | | | | | | 1 | 1 | | | | | | | 2 |
| Gangrene, . . . . . | 1 | 1 | | 1 | | | 1 | | | | | | 1 | | | | | | | 2 |
| Gout, . . . . . . . | 2 | | | | | | | | | | | | | | | | 1 | 1 | | 2 |
| Hemorrhage, . . . . | 5 | 8 | 1 | 3 | 1 | | 1 | 2 | | | 1 | 3 | 1 | 4 | | | | | | 13 |
| Inanition, . . . . . | 9 | 14 | 7 | 11 | 16 | 2 | | | | | | | | 2 | | 3 | | | | 23 |
| Inflammation, . . . . | 4 | 3 | 1 | 3 | 2 | | | 1 | | 1 | 1 | 1 | 1 | | | | | | | 7 |
| " Throat, . | | 2 | | 2 | 2 | | | | | | | | | | | | | | | 2 |
| Malformation, . . . . | 1 | 1 | 1 | 1 | 2 | | | | | | | | | | | | | | | 2 |
| Marasmus, . . . . . | 41 | 47 | 40 | 45 | 50 | 20 | 11 | 2 | 2 | | | 1 | | | | 2 | | | | 88 |
| Mortification, . . . . | | 1 | | | | | | | | | 1 | | | | | | | | | 1 |
| Pyæmia, . . . . . | 1 | | | | | | | | | | | | 1 | | | | | | | 1 |
| Scirrhus, . . . . . | | 2 | | | | | | | | | | 1 | | 1 | | | | | | 2 |
| Scrofula, . . . . . | 13 | 10 | 12 | 10 | 13 | 2 | 3 | 2 | 1 | 1 | 1 | | | | | | | | | 23 |
| Sore Throat, . . . . | 1 | 1 | 1 | 1 | 1 | | 1 | | | | | | | | | | | | | 2 |
| Tabes Mesenterica, . . | 5 | 2 | 5 | | 2 | 1 | 1 | | 1 | | 2 | | | | | | | | | 7 |
| Tumor, . . . . . . | 1 | 2 | 1 | | | | | | 1 | | | | 1 | | 1 | | | | | 3 |
| Ulceration, . . . . . | | 1 | | 1 | | 1 | | | | | | | | | | | | | | 1 |
| " Leg, . . . | | 1 | | | | | | | | | | | | | 1 | | | | | 1 |
| " Throat, . . | 2 | | 2 | | | | 2 | | | | | | | | | | | | | 2 |
| | 195 | 201 | 131 | 115 | 139 | 31 | 43 | 21 | 7 | 5 | 12 | 20 | 20 | 32 | 19 | 34 | 9 | 4 | | 396 |

3. *Nervous Diseases.*

| | Male. | Female. | Boys. | Girls. | Under 1 yr. | 1 to 2. | 2 to 5. | 5 to 10. | 10 to 15. | 15 to 20. | 20 to 30. | 30 to 40. | 40 to 50. | 50 to 60. | 60 to 70. | 70 to 80. | 80 to 90. | 90 to 100. | 100 to 110. | Total. |
|---|---|---|---|---|---|---|---|---|---|---|---|---|---|---|---|---|---|---|---|---|
| Apoplexy, . . . . . | 18 | 14 | 1 | 2 | 2 | | 1 | | | | 3 | 2 | 6 | 5 | 9 | 3 | 1 | | | 32 |
| Coma, . . . . . . | 1 | | | | | | | | | | | | | | | 1 | | | | 1 |
| Concussion of Brain, . | 4 | | 1 | | | | 1 | | | | | 1 | 2 | | | | | | | 4 |
| Congestion of Brain, . | 36 | 23 | 16 | 18 | 16 | 5 | 5 | 6 | | 2 | 2 | 8 | 5 | 6 | 3 | 1 | | | | 59 |
| Convulsions, . . . . | 78 | 63 | 73 | 62 | 79 | 31 | 14 | 7 | 3 | 1 | 1 | | 1 | 2 | 2 | | | | | 141 |
| Cramp, . . . . . . | 2 | 2 | 1 | 2 | 1 | 1 | | | | 1 | 1 | | | | | | | | | 4 |
| Disease of Brain, . . | 19 | 14 | 13 | 12 | 12 | 6 | 2 | 5 | | | | 4 | 2 | 1 | | 1 | | | | 33 |
| Dropsy of Brain, . . . | 36 | 22 | 36 | 21 | 20 | 21 | 15 | 1 | | | 1 | | | | | | | | | 58 |
| Effusion on Brain, . . | 17 | 13 | 13 | 11 | 9 | 5 | 6 | 4 | | | 1 | | 1 | 2 | 1 | 1 | | | | 30 |
| Epilepsy, . . . . . | 6 | 9 | 3 | 5 | 4 | 1 | | 1 | | 2 | 1 | 2 | 2 | 2 | | | | | | 15 |
| Inflammation of Brain, | 32 | 39 | 31 | 36 | 23 | 12 | 18 | 11 | 2 | 1 | 1 | | 3 | | | | | | | 71 |
| Mania, . . . . . . | 1 | 2 | | | | | | | | | | 2 | | 1 | | | | | | 3 |
| Mania-a-Potu, . . . | 8 | | | | | | | | | | | 2 | 3 | 2 | 1 | | | | | 8 |
| Palsy, . . . . . . | 8 | 13 | 1 | | | | 1 | | | | 2 | | 1 | 2 | 1 | 11 | 3 | | | 21 |
| Softening of Brain, . . | 2 | 1 | 1 | | | 1 | | | | | | | 1 | | 1 | | | | | 3 |
| Tetanus, . . . . . | 1 | | | | | | | | | | | | 1 | | | | | | | 1 |
| Trismus, . . . . . | 1 | 1 | 1 | | 1 | | | | | | | | 1 | | | | | | | 2 |
| | 270 | 216 | 191 | 169 | 167 | 83 | 63 | 35 | 5 | 7 | 13 | 21 | 29 | 23 | 18 | 18 | 4 | | | 486 |

4. *Organs of Respiration.*

| | Male. | Female. | Boys. | Girls. | Under 1 yr. | 1 to 2. | 2 to 5. | 5 to 10. | 10 to 15. | 15 to 20. | 20 to 30. | 30 to 40. | 40 to 50. | 50 to 60. | 60 to 70. | 70 to 80. | 80 to 90. | 90 to 100. | 100 to 110. | Total. |
|---|---|---|---|---|---|---|---|---|---|---|---|---|---|---|---|---|---|---|---|---|
| Asthma, . . . . . | 6 | 5 | 4 | | 1 | 1 | 1 | 1 | | | | 2 | | 3 | 1 | 1 | | | | 11 |
| Congestion of Lungs, . | 12 | 17 | 10 | 11 | 13 | 1 | 5 | 2 | | | 3 | 2 | 1 | | 1 | 1 | | | | 29 |
| Consumption of Lungs, | 198 | 209 | 33 | 47 | 9 | 11 | 10 | 4 | 7 | 39 | 125 | 89 | 51 | 33 | 21 | 6 | 2 | | | 407 |
| Disease of Chest, . . | 2 | 2 | 1 | 1 | 1 | | 1 | | | | | 1 | | | 1 | | | | | 4 |
| " Lungs, . . | 4 | 14 | 2 | 6 | 4 | 2 | 1 | | 1 | | 1 | 1 | 3 | 2 | 1 | 1 | 1 | | | 18 |
| Dropsy of Chest, . . | 11 | 3 | 7 | 2 | | | 6 | 3 | | | 1 | | 1 | | 2 | | 1 | | | 14 |
| Effusion on Chest, . . | 1 | | 1 | | | | | | 1 | | | | | | | | | | | 1 |
| " Lungs, . | | 2 | | | | | | | | | | | 1 | | 1 | | | | | 2 |
| Hemr'ge from Lungs, | 4 | 4 | 2 | 1 | 2 | | | | | 1 | 2 | 1 | | | 1 | | 1 | | | 8 |
| Inflam'n of Bronchi, . | 45 | 27 | 37 | 24 | 32 | 13 | 14 | 2 | | | 1 | 2 | 4 | 2 | | 1 | 1 | | | 72 |
| " Chest, . . | 1 | 2 | 1 | 1 | 1 | | 1 | | | | | 1 | | | | | | | | 3 |
| " Larynx, . | 4 | 6 | 3 | 5 | | 5 | 1 | 1 | 1 | | 1 | | 1 | | | | | | | 10 |
| " Lungs, . | 94 | 81 | 63 | 56 | 36 | 34 | 35 | 6 | 1 | 7 | 6 | 15 | 9 | 13 | 5 | 6 | 2 | | | 175 |
| " Pleura, . | | 1 | | 1 | | 1 | | | | | | | | | | | | | | 1 |
| " Tonsils, . | 1 | 4 | 1 | 2 | 2 | | | 1 | | | | 1 | | | | 1 | | | | 5 |
| " Trachea, . | 4 | | 4 | | | 2 | 1 | 1 | | | | | | | | | | | | 4 |
| | 387 | 377 | 169 | 157 | 101 | 70 | 76 | 21 | 11 | 47 | 140 | 115 | 71 | 53 | 34 | 17 | 8 | | | 764 |

5. *Organs of Circulation.*

| | Males. | Females. | Boys. | Girls. | Und'r 1 yr. | 1 to 2. | 2 to 5. | 5 to 10. | 10 to 15. | 15 to 20. | 20 to 30. | 30 to 40. | 40 to 50. | 50 to 60. | 60 to 70. | 70 to 80. | 80 to 90. | 90 to 100. | 100 to 110. | Total. |
|---|---|---|---|---|---|---|---|---|---|---|---|---|---|---|---|---|---|---|---|---|
| Anæmia, . . . . . . . | 4 | 1 | 2 | 1 | 2 | | 1 | | | | | 1 | | | 1 | | | | | 5 |
| Aneurism, . . . . . . | 1 | | 1 | | | | | 1 | | | | | | | | | | | | 1 |
| " Aorta, . . . | 1 | | | | | | | | | | | | | | 1 | | | | | 1 |
| Angina Pectoris, . . . | 1 | | | | | | | | | | | | | | | 1 | | | | 1 |
| Disease of Heart, . . . | 26 | 23 | 11 | 9 | 3 | 1 | 4 | 4 | 6 | 2 | 5 | 5 | 7 | 4 | 6 | 1 | 1 | | | 49 |
| Dropsy of Heart, . . . | 1 | 1 | 1 | | | | 1 | | | | | | | | | 1 | | | | 2 |
| Enlargement of Heart, . | 5 | 5 | 1 | 3 | 1 | | 2 | | | 1 | 1 | | 1 | 4 | | | | | | 10 |
| Inflammation of " . | 1 | 3 | | | | | | | | | 1 | | 1 | 1 | | 1 | | | | 4 |
| Softening of Heart, . . | 1 | | | | | | | | | | | | | | 1 | | | | | 1 |
| | 41 | 33 | 16 | 13 | 6 | 1 | 8 | 5 | 6 | 3 | 7 | 6 | 9 | 9 | 9 | 4 | 1 | | | 74 |

6. *Digestive Organs.*

| | Male. | Female. | Boys. | Girls. | Under 1 yr. | 1 to 2. | 2 to 5. | 5 to 10. | 10 to 15. | 15 to 20. | 20 to 30. | 30 to 40. | 40 to 50. | 50 to 60. | 60 to 70. | 70 to 80. | 80 to 90. | 90 to 100. | 100 to 110. | Total. |
|---|---|---|---|---|---|---|---|---|---|---|---|---|---|---|---|---|---|---|---|---|
| Abscess of Liver, . . . | 1 | | 1 | | | | | | 1 | | | | | | | | | | | 1 |
| Cancer of Stomach, . . | 1 | | | | | | | | | | | | | | 1 | | | | | 1 |
| Cancrum Oris, . . . . | 3 | 5 | 3 | 5 | 1 | | 6 | 1 | | | | | | | | | | | | 8 |
| Cirrhosis Liver, . . . | 1 | | | | | | | | | | | | | | 1 | | | | | 1 |
| Colic, . . . . . . . | 2 | 1 | 1 | | | | 1 | | | | | 1 | | | 1 | | | | | 3 |
| Congestion of Liver, . . | 2 | 1 | | 1 | 1 | | | | | | 1 | | | | 1 | | | | | 3 |
| Disease of Liver, . . . | | 3 | | 2 | 1 | | | 1 | | | | | | | 1 | | | | | 3 |
| " Stom. & Bow., | 4 | 1 | 2 | 1 | 1 | 1 | 1 | | | | | | | 1 | | 1 | | | | 5 |
| Hem'age from Stomach, | 2 | | 1 | | | | | | 1 | | | | 1 | | | | | | | 2 |
| Hernia, Strangulated, . | 1 | | | | | | | | | | 1 | | | | | | | | | 1 |
| " Umbilical, . . | | 1 | | | | | | | | | | | | | | 1 | | | | 1 |
| Inflamm'n of Liver, . . | 2 | 2 | 1 | | 1 | | | | | | | | | 1 | 1 | 1 | | | | 4 |
| Inflamm'n of Peritoneum, | 1 | 22 | 1 | 2 | | | 1 | | 1 | 1 | 9 | 5 | 3 | 1 | 2 | | | | | 23 |
| " Stom. and Bow., | 37 | 33 | 22 | 16 | 13 | 5 | 9 | 7 | 3 | 1 | 8 | 6 | 5 | 9 | 3 | | 1 | | | 70 |
| Intussusception, . . . | 2 | 1 | 2 | | 2 | | | | | | | 1 | | | | | | | | 3 |
| Jaundice, . . . . . | 1 | 3 | 1 | 1 | 2 | | | | | | | | 1 | 1 | | | | | | 4 |
| Perforation of Intestines, | 1 | | 1 | | | | 1 | | | | | | | | | | | | | 1 |
| Scirrhus of Liver, . . | | 1 | | | | | | | | | | | | 1 | | | | | | 1 |
| " Stomach, . | 3 | 1 | | 1 | 1 | | | | | | | | | | | 2 | 1 | | | 4 |
| Sore Mouth, . . . . | | 1 | | 1 | 1 | | | | | | | | | | | | | | | 1 |
| Teething, . . . . . | 6 | 1 | 6 | 1 | 3 | 2 | 2 | | | | | | | | | | | | | 7 |
| Ulceration of Intestines, | 2 | 1 | 1 | 1 | 1 | | | | 1 | | | | | | 1 | | | | | 3 |
| Worms, . . . . . . | | 1 | | 1 | | 1 | | | | | | | | | | | | | | 1 |
| | 72 | 79 | 43 | 33 | 28 | 9 | 21 | 9 | 7 | 2 | 19 | 13 | 10 | 14 | 12 | 5 | 2 | | | 151 |

7. *Urinary Organs.*

| | Male. | Female. | Boys. | Girls. | Under 1 yr. | 1 to 2. | 2 to 5. | 5 to 10. | 10 to 15. | 15 to 20. | 20 to 30. | 30 to 40. | 40 to 50. | 50 to 60. | 60 to 70. | 70 to 80. | 80 to 90. | 90 to 100. | 100 to 110. | Total. |
|---|---|---|---|---|---|---|---|---|---|---|---|---|---|---|---|---|---|---|---|---|
| Albuminuria, . . . . | 1 | | | | | | | | | | 1 | | | | | | | | | 1 |
| Diabetes, . . . . . . | 3 | 1 | 1 | | 1 | | | | | | 1 | 1 | | | | 1 | | | | 4 |
| Disease of Bladder, . . | 1 | | | | | | | | | | | | | | 1 | | | | | 1 |
| " Kidneys, . . | 2 | 1 | | 1 | | | | | 1 | | | 1 | | | | | | 1 | | 3 |
| Inflmm'n of Kidneys, . . | 3 | | 1 | | | | | 1 | | | 1 | | | 1 | | | | | | 3 |
| Rupture of Urethra, . . | 1 | | | | | | | | | | | | 1 | | | | | | | 1 |
| Suppression of Urine, . | 1 | | | | | | | | | | | | | | | | | 1 | | 1 |
| | 12 | 2 | 2 | 1 | 1 | | | 1 | 1 | | 3 | 2 | 1 | 1 | 1 | 1 | | 2 | | 14 |

8. *Organs of Generation.*

| | Male. | Female. | Boys. | Girls. | Under 1 yr. | 1 to 2. | 2 to 5. | 5 to 10. | 10 to 15. | 15 to 20. | 20 to 30. | 30 to 40. | 40 to 50. | 50 to 60. | 60 to 70. | 70 to 80. | 80 to 90. | 90 to 100. | 100 to 110. | Total. |
|---|---|---|---|---|---|---|---|---|---|---|---|---|---|---|---|---|---|---|---|---|
| Cancer of Uterus, . . . | | 3 | | | | | | | | | | | | | 2 | 1 | | | | 3 |
| Childbed, . . . . . . . | | 2 | | | | | | | | | 2 | | | | | | | | | 2 |
| Chlorosis, . . . . . | | 1 | | 1 | | | | | | 1 | | | | | | | | | | 1 |
| Disease of Ovaries, . . | | 2 | | | | | | | | | 2 | | | | | | | | | 2 |
| Disease of Uterus, . . . | | 2 | | | | | | | | | 1 | 1 | | | | | | | | 2 |
| Hem'age from Uterus, . | | 2 | | | | | | | | | 1 | 1 | | | | | | | | 2 |
| Inflamm'n of Uterus, . . | | 5 | | | | | | | | | 2 | 1 | 1 | 1 | | | | | | 5 |
| Phlegmasia Dolens, . . | | 1 | | | | | | | | | 1 | | | | | | | | | 1 |
| Puerperal Convulsions, . | | 1 | | | | | | | | | 1 | | | | | | | | | 1 |
| " Fever, . . . | | 33 | | 6 | | | | | | 6 | 18 | 8 | 1 | | | | | | | 33 |
| Ulceration of Uterus, . . | | 1 | | | | | | | | | | 1 | | | | | | | | 1 |
| | | 53 | | 7 | | | | | | 7 | 28 | 12 | 2 | 1 | 2 | 1 | | | | 53 |

9. *Organs of Locomotion.*

| | Male. | Female. | Boys. | Girls. | Under 1 yr. | 1 to 2. | 2 to 5. | 5 to 10. | 10 to 15. | 15 to 20. | 20 to 30. | 30 to 40. | 40 to 50. | 50 to 60. | 60 to 70. | 70 to 80. | 80 to 90. | 90 to 100. | 100 to 110. | Total. |
|---|---|---|---|---|---|---|---|---|---|---|---|---|---|---|---|---|---|---|---|---|
| Caries, . . . . . . . | 1 | 1 | | 1 | | | 1 | | | | | 1 | | | | | | | | 2 |
| Disease of Hip, . . . . | 1 | | 1 | | | | | 1 | | | | | | | | | | | | 1 |
| Disease of Spine, . . . | 2 | 1 | 2 | 1 | | 1 | | 1 | 1 | | | | | | | | | | | 3 |
| Rheumatism, . . . . | 3 | 3 | 1 | 2 | ] | | | | 1 | 1 | 1 | 1 | 1 | | | | | | | 6 |
| | 7 | 5 | 4 | 4 | 1 | 1 | 1 | 2 | 2 | 1 | 1 | 2 | 1 | | | | | | | 12 |

10. *Integumentary System.*

| | | | | | | | | | | | | | | | | | | | | |
|---|---|---|---|---|---|---|---|---|---|---|---|---|---|---|---|---|---|---|---|---|
| Purpura, . . . . . . . | | 1 | | 1 | | | | 1 | | | | | | | | | | | | 1 |

11. *Old Age.*

| | | | | | | | | | | | | | | | | | | | | |
|---|---|---|---|---|---|---|---|---|---|---|---|---|---|---|---|---|---|---|---|---|
| Old Age, . . . . . . . | 16 | 29 | | | | | | | | | | | | | 2 | 12 | 25 | 5 | 1 | 45 |

12. *External Causes.*

| | Male. | Female. | Boys. | Girls. | Under 1 yr. | 1 to 2. | 2 to 5. | 5 to 10. | 10 to 15. | 15 to 20. | 20 to 30. | 30 to 40. | 40 to 50. | 50 to 60. | 60 to 70. | 70 to 80. | 80 to 90. | 90 to 100. | 100 to 110. | Total. |
|---|---|---|---|---|---|---|---|---|---|---|---|---|---|---|---|---|---|---|---|---|
| Asphyxia, . . . . . | 5 | 4 | 5 | 3 | 6 | | 1 | | 1 | | 1 | | | | | | | | | 9 |
| Burns, . . . . . . | 8 | 6 | 4 | 1 | 1 | 1 | 3 | | | | 4 | 3 | | 1 | | | | 1 | | 14 |
| Casualties, . . . . . | 16 | 5 | 2 | 3 | 3 | 1 | | | 1 | | 4 | 5 | 2 | 2 | 1 | 2 | | | | 21 |
| Drowned, . . . . . . | 3 | | 1 | | | | 1 | | | | 1 | 1 | | | | | | | | 3 |
| Exhaustion, . . . . . | 3 | 1 | 2 | 1 | 2 | 1 | | | | | | | | | | | 1 | | | 4 |
| Exposure, . . . . . | 2 | 2 | | 1 | | | 1 | | | | | 1 | 1 | | | 1 | | | | 4 |
| Fracture, . . . . . . | 2 | | | | | | | | | | | | 2 | | | | | | | 2 |
| " Pelvis, . . . | 1 | | | | | | | | | | | 1 | | | | | | | | 1 |
| " Skull, . . . | 3 | 1 | 2 | | | 1 | | | | 1 | | | | 1 | 1 | | | | | 4 |
| Intemperance, . . . . | 2 | 2 | | | | | | | | | 2 | | 1 | 1 | | | | | | 4 |
| Suicide, . . . . . . | 2 | 2 | | | | | | | | | 1 | 1 | 1 | | 1 | | | | | 4 |
| Suffocation, . . . . . | 3 | 1 | 3 | 1 | 3 | 1 | | | | | | | | | | | | | | 4 |
| Violence, . . . . . . | 4 | | | | | | | | | | 3 | | | 1 | | | | | | 4 |
| | 54 | 24 | 19 | 10 | 15 | 5 | 6 | | 2 | 1 | 16 | 12 | 7 | 6 | 3 | 3 | 1 | 1 | | 78 |
| Still Born, . . . . . | 71 | 58 | 71 | 58 | 129 | | | | | | | | | | | | | | | 129 |
| Unknown, . . . . . | 48 | 37 | 24 | 18 | 34 | 1 | 6 | | | 1 | 9 | 6 | 9 | 8 | 9 | 2 | | | | 85 |

TABLE No. 3.

*Deaths for the First Quarter of* 1857, *at fifteen distinct periods of life.*

| | |
|---|---|
| Under 1 year, . . . . . . . . . | 602 |
| 1 to 2, . . . . . . . . . | 331 |
| 2 to 5, . . . . . . . . . | 500 |
| 5 to 10, . . . . . . . . . | 206 |
| 10 to 15, . . . . . . . . . | 63 |
| 15 to 20, . . . . . . . . . | 82 |
| 20 to 30, . . . . . . . . . | 280 |
| 30 to 40, . . . . . . . . . | 229 |
| 40 to 50, . . . . . . . . . | 174 |
| 50 to 60, . . . . . . . . . | 158 |
| 60 to 70, . . . . . . . . . | 117 |
| 70 to 80, . . . . . . . . . | 107 |
| 80 to 90, . . . . . . . . . | 51 |
| 90 to 100, . . . . . . . . . | 12 |
| 100 to 110, . . . . . . . . . | 1 |
| | 2,913 |
| Still born, . . . . . | 129 |
| Total, . . . . . . | 3,042 |

Of the above deaths, 153 were from the Blockley Almshouse; 139 from the colored population; and 14 from the country, as follows:

| | January. | February. | March. | Total. |
|---|---|---|---|---|
| Almshouse, . . . . | 53 | 51 | 49 | 153 |
| Blacks, . . . . . | 45 | 48 | 46 | 139 |
| Country, . . . . . | 6 | 3 | 5 | 14 |
| | 104 | 102 | 100 | 306 |

TABLE No. 4.

The following table furnishes the number of deaths for each week during the quarter, together with the sexes, the adults, and children, with their sexes.

| | Male. | Female. | Boys. | Girls. | Adults. | Children. | Total. | Monthly Totals. |
|---|---|---|---|---|---|---|---|---|
| 1857. From Jan. 3d to 10th, . . | 136 | 128 | 96 | 71 | 97 | 167 | 264 | |
| " Jan. 10th to 17th, . | 153 | 140 | 102 | 98 | 93 | 200 | 293 | |
| " Jan. 17th to 24th, . | 168 | 123 | 116 | 75 | 100 | 191 | 291 | |
| " Jan. 24th to 31st, . . | 148 | 143 | 95 | 85 | 111 | 180 | 291 | 1139 |
| " Jan. 31st to Feb. 7th, | 162 | 135 | 113 | 82 | 102 | 195 | 297 | |
| " Feb. 7th to 14th, . . | 114 | 120 | 77 | 77 | 80 | 154 | 234 | |
| " Feb. 14th to 21st, . . | 123 | 127 | 80 | 79 | 91 | 159 | 250 | |
| " Feb. 21st to 28th, . . | 126 | 88 | 77 | 56 | 81 | 133 | 214 | 995 |
| " Feb. 28th to Mar. 7th, | 120 | 100 | 75 | 57 | 88 | 132 | 220 | |
| " Mar. 7th to 14th, . . | 121 | 104 | 78 | 59 | 88 | 137 | 225 | |
| " Mar. 14th to 21st, . | 127 | 135 | 77 | 73 | 112 | 150 | 262 | |
| " Mar. 21st to 28th, . | 97 | 104 | 53 | 62 | 86 | 115 | 201 | 908 |
| | 1595 | 1447 | 1039 | 874 | 1129 | 1913 | 3042 | 3042 |

